# Surgical Strategy for Multiple Nasal Basal Cell Carcinomas: Narrow-Margin Excision With Secondary Intention Healing

**DOI:** 10.7759/cureus.96357

**Published:** 2025-11-08

**Authors:** Yuto Yamamura, Kazuyasu Fujii, Chisa Nakashima, Shunya Usui, Kazutoshi Nishimura, Atsushi Otsuka

**Affiliations:** 1 Dermatology, Kindai University, Osaka, JPN; 2 Dermatology, Kindai University Hospital, Osaka, JPN

**Keywords:** basal cell carcinoma, multiple lesions, narrow-margin excision, nose, secondary intention healing

## Abstract

Basal cell carcinoma (BCC) is the most common type of skin cancer, often occurring on the nose, where reconstruction is particularly challenging. Managing multiple nasal BCCs presents additional difficulties because multiple surgical defects within the same aesthetic subunit can limit flap mobility and compromise cosmetic outcomes. We report a case of two histopathologically confirmed nodular BCCs on the nasal dorsum treated with different surgical strategies tailored to each lesion. The left lesion, which required flap reconstruction, was excised with a 2-mm margin to ensure complete tumor removal. The right lesion, smaller and sharply demarcated, was excised with a 0.5-mm margin and allowed to heal by secondary intention. Histopathological examination confirmed tumor-free margins in both specimens. The open wound on the right side was completely epithelialized within three weeks, while the rotation flap on the left cheek healed uneventfully. At six months postoperatively, both sites demonstrated excellent cosmetic outcomes with minimal scarring and no recurrence. This case highlights that individualized surgical planning based on lesion characteristics and histopathologic subtype ensures both oncologic safety and aesthetic preservation. Narrow-margin excision with secondary intention healing represents a practical and minimally invasive option for small, well-defined nasal BCCs.

## Introduction

Multiple basal cell carcinomas (BCCs) are not uncommon even in immunocompetent individuals, with 25-55% of patients reported to have two or more lesions [1]. Recent population-based data also indicate that nearly 40% of patients with BCC develop multiple lesions during their lifetime [2]. Among these, the nose is the most frequently affected site, accounting for approximately 22-27% of multiple BCC cases [2].

The nasal region presents both anatomical and aesthetic challenges for reconstruction [3]. In patients with multiple BCCs, the presence of several defects within the same subunit can restrict flap mobility and lead to problems such as limited skin redundancy or interference between adjacent flaps [4]. Therefore, an excision strategy that anticipates reconstruction is crucial in managing multiple nasal BCCs.

BCC is typically locally invasive but rarely metastasizes [5], and well-demarcated lesions allow for safe use of narrow excision margins without compromising oncologic control [6].

Herein, we report a case of two nodular BCCs occurring bilaterally on the nasal dorsum. By tailoring the excision margins according to tumor size and selecting narrow-margin excision with secondary intention healing (SIH) for the smaller lesion, we achieved both oncologic control and favorable cosmetic outcomes. This case highlights the practicality and rationale of such a tailored surgical approach for multiple nasal BCCs.

## Case presentation

**Figure 1:**
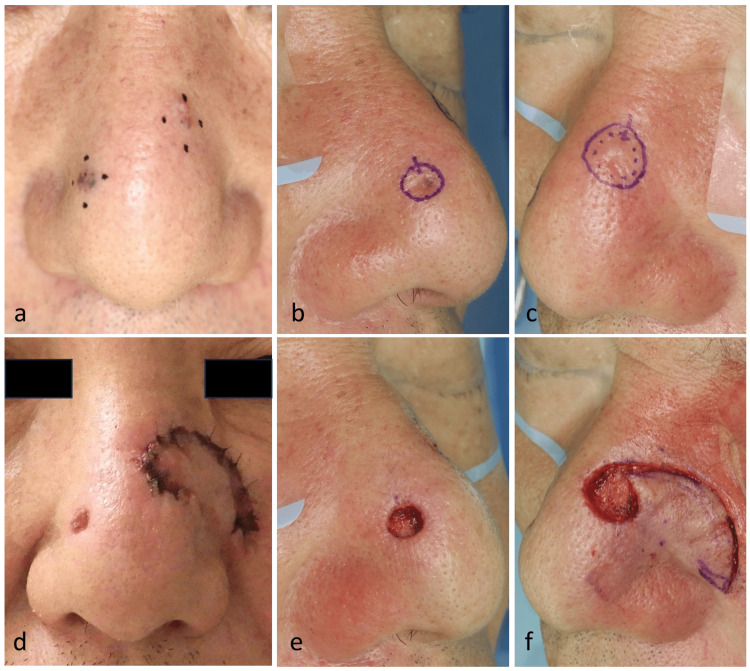
Preoperative and intraoperative findings of multiple basal cell carcinomas (BCCs) on the nose. A: Preoperative appearance. Black papules were observed on both sides of the nasal dorsum. Biopsy confirmed nodular BCC in both lesions. B: Surgical design for the right nasal lesion. The excision line was planned with a 0.5-mm margin based on the clinically defined border. C: Surgical design for the left nasal lesion. A 2-mm margin was planned to ensure complete tumor removal. D: Immediate postoperative view. The right defect was left open for secondary intention healing, and the left defect was reconstructed with a rotation flap from the left cheek. No nasal deformity was observed. E: Intraoperative view of the right nasal lesion. The tumor was excised with a 0.5-mm margin, and the defect was left as an open wound. F: Intraoperative view of the left nasal lesion. After tumor excision, a rotation flap was elevated from the left cheek and transposed to close the defect.

A man in his sixties presented with a black papule on the left nasal dorsum that had gradually enlarged over three years. During evaluation at a previous clinic, a similar lesion was also noted on the right nasal dorsum, and he was referred to our department for further assessment and treatment. He was a retired office worker with no history of significant occupational or recreational ultraviolet exposure, and no relevant environmental exposure such as radiation or toxic substances. There was also no family history of skin cancer. Physical examination revealed black nodules on both sides of the nasal dorsum. Biopsy specimens confirmed nodular BCC in both lesions. The tumors measured 7 mm on the right and 8 mm on the left (Fig. 1a).

To ensure a negative margin, the left lesion was excised with a 2 mm margin, and reconstruction was performed using a rotation flap from the left cheek. Considering cosmetic preservation, the right lesion was excised with a 0.5 mm margin, and the resulting defect was left open for SIH (Fig. 1b-1f). Both procedures were performed under local anesthesia with 1% lidocaine.

**Figure 2:**
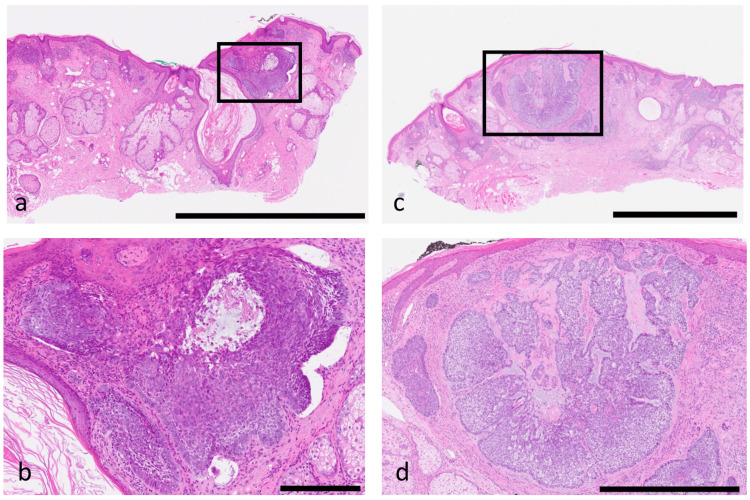
Histopathological findings of BCCs on the nose (hematoxylin–eosin stain). A: Low-power view of the right nasal lesion. Multiple tumor nests with well-defined borders are observed within the dermis, showing predominantly nodular growth. The surgical margins are free of tumor cells, and the nests are clearly demarcated from the surrounding fibrous stroma (scale bar = 2.5 mm). B: High-power view of the same lesion. The tumor nests consist of small basaloid cells with peripheral palisading and distinct retraction artifacts between the tumor and stroma (scale bar = 250 µm). C: Low-power view of the left nasal lesion. Nodular tumor nests are confined to the mid-dermis, with tumor-free margins at a sufficient distance. The invasion is superficial, without extension into the subcutaneous fat layer (scale bar = 2.5 mm). D: High-power view of the same lesion. The tumor consists of uniform basaloid cells accompanied by mild stromal fibrosis. Peripheral palisading is evident along the tumor nests, consistent with the typical morphology of nodular BCC (scale bar = 1 mm).

Histopathologic examination revealed nodular-type BCC confined to the dermis, with tumor-free margins in both specimens (Fig. 2a-2d).

The open wound on the right side re-epithelialized within approximately three weeks, and the left rotation flap healed uneventfully. Postoperative care consisted of applying topical gentamicin ointment to the open wound until complete epithelialization, with regular outpatient follow-up during the healing period. Mohs micrographic surgery was not considered, as both lesions were well-defined and could be completely excised with conventional surgery; moreover, Mohs surgery is not routinely performed for BCCs in Japan.

**Figure 3:**
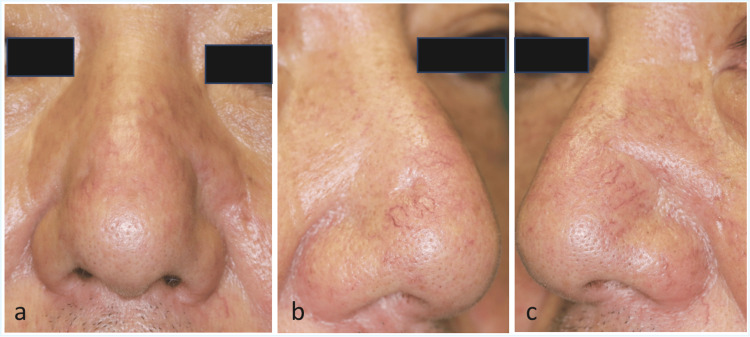
Postoperative appearance at six months. A: Frontal view showing well-preserved nasal contour without any obvious deformity. B: Right oblique view. The defect on the right nasal ala has epithelialized through secondary intention healing, leaving only a faint scar with satisfactory cosmetic healing. C: Left oblique view. The rotation flap on the left nasal ala maintains a natural contour with minimal scarring. No evidence of recurrence is observed on either side.

At six months postoperatively, both sides had healed with minimal scarring, maintaining normal nasal contour and achieving a satisfactory cosmetic outcome (Fig. 3a-3c). No recurrence has been observed.

## Discussion

In general, a predetermined safety margin is recommended for the excision of BCCs. However, a BCC is characterized by local invasiveness but extremely rare metastasis [5]. Therefore, when histologically tumor-free margins can be ensured, narrow-margin excision is considered acceptable [6].

In the present case, both lesions on the nasal dorsum were of the nodular type with clinically well-defined borders. To achieve a balance between complete tumor removal and cosmetic preservation, different surgical strategies were applied to each lesion.

The left lesion was excised with a 2-mm margin to ensure negative margins, as flap reconstruction was planned. Recent studies have reported that excision with a 2-mm margin achieves histologically clear margins in more than 95% of well-demarcated pigmented BCCs [7], supporting the appropriateness of this approach.

By contrast, the right lesion was excised with a reduced margin of 0.5 mm. This decision was based on the sharply defined clinical border and the histopathological subtype of nodular type BCC, which suggested a high likelihood of complete excision, and the expectation that minimizing the defect size would allow favorable healing by secondary intention. Indeed, SIH has been reported to yield high patient satisfaction in terms of both cosmetic and functional outcomes following nasal tumor excision [8,9]. Furthermore, defects with a diameter of less than 1 cm and confined above the superficial fat layer are known to achieve excellent cosmetic results regardless of the nasal subunit [10].

As a result, the right-sided lesion was completely excised with tumor-free margins, and the defect size was limited to approximately 8 mm in diameter, leading to an excellent cosmetic outcome following SIH. Moreover, managing the right lesion with narrow-margin excision and SIH minimized interference with the adjacent rotation flap on the left side and helped prevent overall nasal distortion.

This case demonstrates that adjusting excision margins and reconstruction methods according to individual lesion characteristics can effectively achieve both oncologic control and aesthetic preservation. In addition, even if the surgical margin of the right lesion had been positive, the open wound would have allowed for straightforward re-excision, making this approach both oncologically sound and pragmatically safe.

Multiple BCCs are relatively common even in immunocompetent individuals. Reported risk factors include advanced age, male sex, Fitzpatrick skin type I-II, a history of chronic ultraviolet exposure, and previous BCCs [1]. Moreover, multiple BCCs may not only occur synchronously but also develop metachronously over time [11].

Therefore, when treating the initial lesion, it is advisable to design a surgical plan that anticipates the possible emergence of future lesions while maintaining reconstructive flexibility. Selecting minimally invasive approaches, such as narrow-margin excision or simple reconstruction methods like SIH, can be beneficial not only for aesthetic preservation but also for maintaining future treatment options. In addition, these approaches generally involve less surgical burden and lower overall cost, which may further support their clinical practicality.

## Conclusions

This case illustrates that tailoring excision strategies for each lesion can achieve both complete tumor control and satisfactory cosmetic outcomes in patients with multiple nasal BCCs. By selecting surgical margins and reconstruction methods based on individual lesion characteristics, such as size, subtype, and anatomical location, clinicians can optimize both oncologic safety and aesthetic preservation. The combination of narrow-margin excision and secondary intention healing for small, well-defined lesions offers a minimally invasive yet effective treatment option, particularly in areas where tissue mobility is limited. This patient-specific, flexible approach may serve as a practical model for managing multiple BCCs in cosmetically sensitive regions like the nose, contributing to improved patient satisfaction and long-term reconstructive planning.
